# Understanding the role of interpersonal violence in assisted partner notification for HIV: a mixed-methods study in refugee settlements in West Nile Uganda

**DOI:** 10.7189/jogh.10.020440

**Published:** 2020-12

**Authors:** Robin E Klabbers, Timothy R Muwonge, Emmanuel Ayikobua, Diego Izizinga, Ingrid V Bassett, Andrew Kambugu, Alexander C Tsai, Miranda Ravicz, Gonnie Klabbers, Kelli N O’Laughlin

**Affiliations:** 1 Faculty of Health, Medicine, and Life Sciences, Maastricht University, Maastricht, the Netherlands; 2 Infectious Diseases Institute, College of Health Sciences, Makerere University, Kampala, Uganda; 3 Department of Medicine, Massachusetts General Hospital, Boston, Massachusetts, USA; 4 Center for Global Health and Mongan Institute, Massachusetts General Hospital, Boston, Massachusetts, USA; 5 Department of Internal Medicine and Pediatrics, Massachusetts General Hospital, Boston, Massachusetts, USA; 6 Department of Health, Ethics and Society, Faculty of Health, Medicine, and Life Sciences, Maastricht University, Maastricht, the Netherlands; 7 Departments of Emergency Medicine and Global Health, University of Washington, Seattle, Washington, USA

## Abstract

**Background:**

Assisted partner notification (APN) for HIV was introduced in refugee settlements in West Nile Uganda in 2018 to facilitate testing of sexual partners. While APN is an effective strategy recommended by the World Health Organization, its safety has not been evaluated in a refugee settlement context in which participants have high prior exposure to interpersonal violence. The extent to which interpersonal violence influences APN utilization and the frequency with which post-APN interpersonal violence occurs remains unknown.

**Methods:**

To explore the relationship between APN and interpersonal violence, a cross-sectional mixed-methods study was conducted at 11 health centers in or near refugee settlements serving refugee and national populations in West Nile Uganda. Routinely collected index client and sexual partner data were extracted from APN registers and semi-structured interviews were conducted with health workers.

**Results:**

Through APN, 1126 partners of 882 distinct index clients were identified. For 8% (75/958) of partners, index clients reported a history of intimate partner violence (IPV). For 20% (226/1126) of partners, index clients were screened for post-APN IPV; 8 cases were reported of which 88% (7/8) concerned partners with whom index clients reported prior history of IPV. In qualitative interviews (N = 32), health workers reported HIV disclosure-related physical, sexual and psychological violence and deprivation or neglect. Incidents of disclosure-related violence against health workers and dependents of index clients were also reported. Fear of disclosure-related violence was identified as a major barrier to APN that prevents index clients from listing sexual partners.

**Conclusions:**

Incidents of interpersonal violence have been reported following HIV-disclosure and fear of interpersonal violence strongly influences APN participation. Addressing HIV perception and stigma may contribute to APN uptake and program safety. Prospective research on interpersonal violence involving index clients and sexual partners in refugee settlements is needed to facilitate safe engagement in APN for this vulnerable population.

Assisted partner notification (APN) is a public health program recommended by the World Health Organization (WHO) in which trained providers assist consenting clients living with HIV (“index clients”) in notifying their sexual partners of potential HIV exposure so that the partners can be tested and linked to care [1]. APN has been highly effective in increasing uptake of HIV testing among sexual partners and in yielding new HIV diagnoses in sub-Saharan Africa (SSA) [2-9]. In many countries in this region however, HIV remains a deeply stigmatized disease and disclosure of living with HIV may elicit negative and even violent reactions [10-14], raising questions regarding the safety of APN for these settings. Apart from representing a serious human rights concern, HIV disclosure-related violence forms a serious threat to public health as it can lead to significant mental health problems [15,16] that undermine adherence to HIV treatment [17-19]. The studies that form the basis of the 2016 WHO Guidelines on HIV self-testing and partner notification [1], along with multiple recent studies evaluating APN [20-25] have reported that social harm following APN participation is rare and have found no association between APN and subsequent intimate partner violence (IPV) (for definitions of the different forms of violence refer to **Table 1** and **Figure 1**). However, the study designs leave various aspects of the issue uninvestigated [2,4,6-9,30].

**Table 1:** Definitions of different types of violence

Term	Abbreviation	Definition
Interpersonal violence		The **intentional use of physical force or power against other persons by an individual or small group of individuals**. Interpersonal violence may be physical, sexual, or psychological (also called emotional violence), and it may involve deprivation and neglect. Acts of interpersonal violence **can be further divided into family or partner violence and community violence [**26**].**
Intimate partner violence	IPV	Behavior **within an intimate relationship** that causes physical, sexual or psychological harm, including acts of physical aggression, sexual coercion, psychological abuse and controlling behaviors [27]*
Gender-based violence	GBV	Any act that is perpetrated against a person’s will and is **based on gender norms and unequal power relationships**. It encompasses threats of violence and coercion. It can be physical, emotional, psychological, or sexual in nature, and can take the form of a denial of resources or access to services [28]†.

*This definition covers violence by both current and former spouses and partners.

†Although the terms *GBV* and *violence against women* are often used interchangeably, GBV can inflict harm on men and boys as well as women and girls.

**Figure 1:**
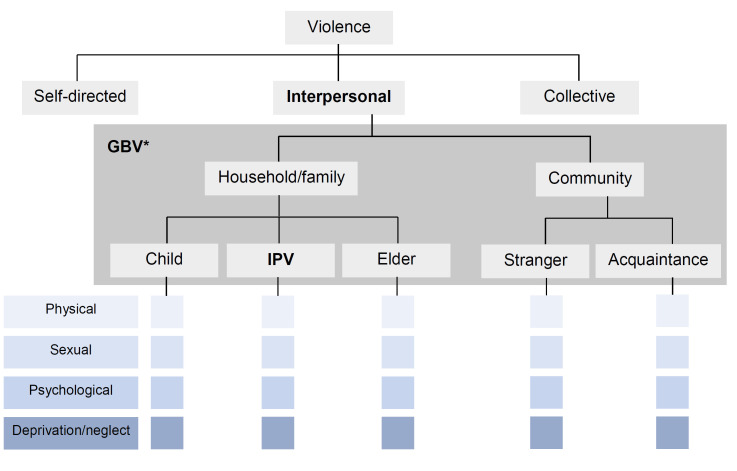
A typology of violence [29]. GBV – gender-based violence, IPV - Intimate partner violence. *When motivated by gender norms and unequal power relationships

In some previous studies, index clients with a recent history of IPV, a known predictor of abuse following HIV disclosure [11,31,32], have been excluded from APN program participation for safety reasons [4,6,22]. In a Kenyan cluster-randomized controlled trial of APN, index clients who had experienced IPV in the previous month were considered to be at a high-risk for post-APN violence and were therefore not considered eligible for study participation leading to the exclusion of 2.1% of potential participants [4]. In another study, index clients were asked to exclude those sexual partners from whom they expected a violent reaction to notification of HIV exposure [33]. Though excluding participants who were deemed to be high-risk for subsequent violence was a logical and likely necessary approach, because of these exclusion criteria, a gap in knowledge exists regarding the safety of APN for people with a heightened risk for post-APN violence, a limitation that is of particular concern for participants in APN programs where these same exclusion criteria are not followed.

An additional limitation of prior work is that while previous APN studies monitored for and reported rates of post-APN violence from sexual partners and reported rates of post-APN IPV [6,7,9], violence perpetrated by other parties was not examined. The lack of data on violence by third parties such as family or community members is of concern for regions of the world where a collectivist culture is dominant [34] in which extended family and community members are highly involved in relationships [35]. This is underscored by studies across Asia, the Middle East and Africa that identify in-laws as frequent instigators of conflict within marital relationships and which demonstrate that family members sometimes engage directly in violent conflict [36-40]. In one such study conducted in Jordan, up to 45% of women reported experiencing interference in their relationship from the wider family, with 26% reporting violence from family members [38], suggesting that monitoring for violence from perpetrators other than intimate partners may be warranted.

The scope of a number of previous studies is further limited by a focus on violence solely as a possible consequence of participation in APN. The threat of violence however, may also be an important factor in shaping APN uptake and the modality of partner notification chosen by index clients. This hypothesis is supported by the indication in several studies that fear of violence is experienced as an important barrier to APN by index clients [22,41].

Understanding how *interpersonal violence* – defined as physical, sexual, or psychological violence, or deprivation and neglect, perpetrated by partners, family, or community members [26] (**Table 1** and **Figure 1**) – relates to APN utilization and safety should be a priority. This is especially urgent in high-risk settings such as refugee settlements where the population has a high prevalence of prior exposure to violence [42-46] and the risk of future violence is heightened [11,31,32].

To gain a better understanding of the role of interpersonal violence in APN, a mixed-methods study was conducted at 11 health centers in or near refugee settlements serving refugee and national populations in West Nile Uganda. We extracted routinely collected data on index clients and their sexual partners from APN registers and conducted semi-structured qualitative interviews with health workers directly involved in HIV testing and counseling services to assess how APN service utilization is influenced by interpersonal violence and whether APN participation is associated with subsequent violence in this high-risk setting. An additional aim of this research was to investigate barriers and facilitators to APN in the refugee context; these findings will be reported in a separate manuscript.

## METHODS

### Study site

This study was conducted at 11 health centers in the West Nile districts Adjumani, Arua, Moyo, and Yumbe that provide care to refugees and Ugandan nationals and were reporting data on APN services to the Infectious Diseases Institute at Makerere University in January 2019. West Nile is a region in northwestern Uganda bordered by the Democratic Republic of the Congo (DRC) and the Republic of South Sudan, populated by a refugee population of over 700 000 [47-49]. Many of these refugees were exposed to violence during the Sudanese civil war in 2013 or experienced violence while fleeing their home country [47,50-52]. The prevalence of HIV among refugees living in West Nile is unknown. Previous research in Uganda and SSA however, has generally shown a lower HIV prevalence among refugees compared to the prevalence among the host population [51,52], which is 3.1% in the West Nile region [53]. In West Nile, APN services were introduced as part of HIV testing and care in 2018 [54]. Since APN initiation in the region, there have been reports of HIV disclosure-related violence and medical staff in refugee settlements have described incidents of starvation and assault of individuals recently diagnosed with HIV.

### APN in Uganda

In Uganda, voluntary APN participation is offered to all newly diagnosed index clients identified through voluntary counseling and testing (VCT), provider-initiated counseling and testing (PICT) and prevention of mother-to-child transmission (PMTCT). APN services are also offered to all previously enrolled index clients with increased risk (index clients not on anti-retroviral therapy [ART], not virally suppressed, or found to have a sexually transmitted disease or a new sexual partner) [54]. As part of APN in Uganda, index clients are asked to list their sexual partners and select one of the following three notification options to notify each partner of possible HIV exposure: “self-notification” in which the index client discloses to their sexual partner(s), “provider notification” in which the health worker discloses to the sexual partner(s) without revealing the identity of the index client, and “assisted notification” which is a combination of the other two options. In assisted notification, index clients are initially given a two-week period to notify their sexual partner(s) and sign a contract agreeing that the health worker may notify the partner(s) if the index client has not notified the partner(s) within the allocated timeframe [54]. In practice, in a high percentage of self-notification cases, index clients do not notify their sexual partners of exposure within the two-week period and health worker-mediated notification is implemented. Although these cases are recorded as “self-notification”, they ultimately default to “assisted notification”.

For each index client who participates in APN, an APN form is filled out in the APN register by a health worker. On the APN form, health workers record the sociodemographic information of the index client and any sexual partners whom the index client identifies. For each sexual partner, the chosen notification option is recorded, as well as the answer “yes/no” to whether there has been a “prior history of violence with this partner”. After APN is completed, the index client is screened for post-APN violence within one month. Whether screening has taken place is reflected by the selection of “yes/no”. The outcome of this screening is recorded by the health worker, who indicates whether the index client has been exposed to violence from an intimate partner using the response options “yes/no”. While the APN form refers to this form of violence as “gender-based violence” (GBV), there is no explicit inquiry into gender norms or power dynamics, and in practice the screening is for intimate partner violence (IPV) (**Table 1** and **Figure 1**). Whether identified sexual partners report to the health facility for HIV testing following notification and the outcome of HIV testing are also recorded on the APN form.

### Study design

In July 2019, a cross-sectional mixed-methods study was conducted in which quantitative and qualitative data were collected concurrently, analyzed independently, and interpreted together, allowing for the corroboration and contextualization of findings [55].

### Data collection and classification of endpoints

#### Quantitative data

Data on index clients and sexual partners routinely collected and recorded by health workers as part of APN were de-identified and extracted from written HIV and APN registers at each health center for all 882 index clients who participated in APN and the 1126 sexual partners they identified from the time of APN initiation at each health center (ranging from December 2017 to January 2019) until the time of this study (July 2019). Extracted data included refugee or Ugandan national status of index clients, the APN notification option chosen to notify sexual partners, the presence of a prior history of IPV, screening for post-APN violence and the occurrence of post-APN IPV. De-identified APN and HIV register data were entered into a secure electronic REDCap database.

#### Qualitative data

Purposive sampling was used to identify 2-3 health workers at each health center based on the assumption that a total of 20-40 interviews would be sufficient to reach data saturation for themes and meta-themes that cut across sites [56-59]. English-speaking health workers, 18 years and older, involved in HIV testing and care were considered eligible. No health workers were excluded based on these eligibility criteria. Written informed consent was obtained from all participants. For each interview participant, limited demographic data were recorded including sex, age, role in APN, and years of work experience. Interviews were semi-structured and were conducted in English by a single interviewer (REK) using an interview guide informed by Weinstein et al.’s Precaution Adoption Process Model (PAPM) [60-62]. The PAPM seeks to distinguish the cognitive stages individuals pass through when adopting health protective behavior and to identify factors governing movement from one stage to the next. For this study, the PAPM was adapted, as done previously by Plotkin et al. [5], to aid in the identification of factors influencing APN utilization by index clients and to consider the different stages sexual partners pass through when deciding to get tested for HIV. These stages were also examined through the lens of interpersonal violence. Throughout the study, the interview guide was iteratively refined based on emerging themes. Interviews were conducted in private at the health center. Participants were compensated UGX20 000 (approximately US$5.40 or EUR€4.90). All interviews were audio-recorded with participants’ permission. De-identified audio files and interview transcripts as well as sociodemographic details of interview participants were uploaded to the REDCap database.

### Data analysis

#### Quantitative data

Data were analyzed using IBM SPSS Statistics (version 25) (IBM Inc, Armonk, NY, USA) [63]. Simple frequencies were used to describe prior history of IPV, whether participants were screened for post-APN IPV, and incidents of post-APN IPV. Cross-tabulations with χ^2^ analysis were used to determine whether there were significant differences in prior IPV exposure between refugees and Ugandan nationals and whether a history of IPV significantly influenced notification option choice. A *P* value <0.05 was considered statistically significant.

#### Qualitative data

Thematic analysis was used to analyze qualitative data [64]. Five interview transcripts were selected and independently reviewed by three researchers (KNO, TRM, REK) using open coding followed by focused coding for recurring ideas and themes. Emerging themes were organized into categories and used to develop an analytic coding framework. The framework was discussed among the three researchers until a consensus was reached. Based on the agreed upon coding framework, a code book was created that was subsequently applied by one researcher (REK) to the remaining transcripts. Following independent analysis, quantitative and qualitative results were integrated, assessing the extent to which these two sets of findings converged or diverged from each other to gain a more in-depth understanding of the role of interpersonal violence in APN.

### Ethical considerations

This study was conducted with ethical oversight from the Makerere University School of Health Sciences Institutional Review Board (SHSREC REF: 2019-033) and support from the Infectious Diseases Institute Scientific Review Committee at Makerere University, Uganda. Approval was obtained from the Ethical Review Committee of the Faculty of Health, Medicine and Life Sciences at Maastricht University, the Netherlands (FHML/GH_2019.046). An exempt status for review was obtained from the Human Subjects Division at the University of Washington, Seattle, Washington, USA (STUDY00007648). Consistent with national guidelines, clearance to conduct the study was also obtained from the Ugandan National Council of Science and Technology (SS 5032).

## RESULTS

### Quantitative results

Since APN initiation, 882 index clients participated in APN and provided information for 1126 sexual partners. The majority of index clients who participated in APN were female (58%) and index clients had an average age of 35 years (minimum [min] 16 years, maximum [max] 76 years, standard deviation [SD] 9.46 years). Sexual partners were predominantly male (54%) and had an average age of 34 years (min 16 years, max 68 years, SD = 9.04).

#### Prior history of IPV

**Figure 2:**
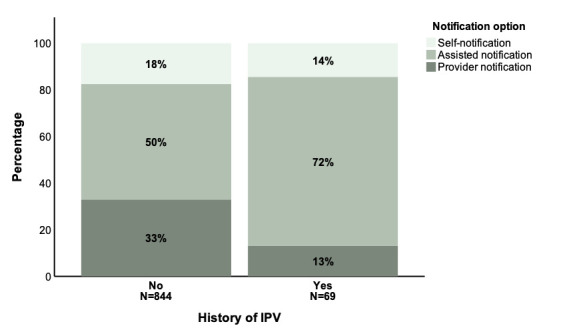
Notification option choice dependent on a presence of a history of IPV (history of IPV no N = 844, history of IPV yes N = 69). IPV – intimate partner violence

Health workers screened index clients for a prior history of IPV for 85% (958/1126) of sexual partners. For 8% (75/958) of these sexual partners who had been evaluated for a prior history of IPV, index clients reported that a prior history of IPV was present. This proportion did not differ significantly between refugees and Ugandan nationals (7% (29/399) vs 9% (38/438), respectively; *P* = 0.56). There was a statistically significant difference in the mode of partner notification (ie, self, assisted or provider notification) chosen by index clients based on whether or not a prior history of IPV was present (**Figure 2**). A greater percentage of index clients opted for assisted notification and a smaller percentage opted for self-notification and provider notification when prior history of IPV was present compared to when no prior history of IPV was present (assisted notification 73% (50/69) vs 50% (418/844), self-notification 14% (10/69) vs 18% (148/844), provider notification 13% (9/69) vs 33% (278/844), respectively; *P* = 0.001).

#### Post-APN violence

Following APN, index clients were screened for post-APN violence in relation to 20% (226/1126) of sexual partners (ie, the question “Index client assessed for post-notification violence [<1 month]?” was filled out with “Yes”). For 17% (195/1126) of sexual partners, there was documentation of whether post-APN IPV occurred (ie, the question “GBV positive?” “Yes/No” was filled out). For the 195 index client and sexual partner pairs for which the question on post-APN IPV was answered, 8 cases of post-APN IPV were recorded, corresponding with a post-APN IPV prevalence of 4% (8/195) among the pairs with this question completed. Of these 8 cases, 7 involved sexual partners for whom a prior history of IPV had been reported and 1 case concerned a relationship for which no prior history of IPV had been reported. Of the 8 cases of post-APN IPV, the notification option selected had been assisted notification for 5 cases and provider notification for 3 cases. None of the index clients who experienced post-APN violence had selected self-notification as their notification option. Post-APN violence was reported for both refugee and Ugandan national index clients (4 vs 3 cases respectively) and regarded both male and female index clients (5 vs 3 cases respectively).

### Qualitative results

A total of 32 health workers involved in HIV testing and care were interviewed about their perspectives on APN and their experiences with the program as they relate to interpersonal violence. Interview participants held a variety of positions at the health center with a mean work experience of 5 years (min 1, max 23 years, SD = 4.50). The mean age of interview participants was 32 years (min 20, max 48 years, SD = 7.52) and the majority of participants were male (53%, 17/32).

#### Fear of interpersonal violence influencing APN participation

Health workers identified fear of interpersonal violence following notification to be one of the main barriers to APN participation for index clients. In West Nile, HIV carries connotations of sexual promiscuity and index clients fear that listing their sexual partners and notifying sexual partners of possible HIV exposure may lead to violent retribution. This fear, along with the fear of additional negative consequences – such as accusations of infidelity, termination of relationships and stigma by the community – causes index clients to refrain from identifying sexual partners, especially non-primary and casual sexual partners. Interview participants considered fear of violence a deterrent especially to women as a result of differences in what is considered culturally acceptable sexual behavior for men and for women.

“In our setting, women are supposed to have one husband, it is men who have very many women… So, it is making women to fear disclosing… They are afraid of violence of course, from the husbands, even the relatives of the husband. Because they will say, ‘You are cheating me, you are’ and even divorce.” *Health worker 54-10-2-002, female.*

This fear of violence that is experienced by index clients exists despite the availability of notification options such as provider notification in which their identity is not revealed to sexual partners. Health workers clarified that this persistent fear of post-APN violence stems from a lack of confidence in the program’s confidentiality, especially when language barriers between health workers and refugee index clients necessitate the involvement of translators.

“So sometimes these translators go to the community and they cause problems. They may even know the community and spread out what is happening here [at the health center]… Confidentiality is sometimes even hard to adhere for the health workers… The testing point it is open, and even the ART clinic itself, people do access all the time, so much as we have confidentiality… it is not maximum.” *Health worker 54-02-2-001, male.*

#### IPV as common and necessitating intervention prior to APN

Health workers reported that in this setting, IPV occurs frequently in relationships and is therefore something that needs to be considered before introducing APN services which might trigger violence. Interview participants described that in West Nile, IPV is commonly seen in the form of GBV and it is strongly linked to power imbalances between men and women.

“The men have a habit of beating up their women, they call it disciplining, but it is beating up women… So you find that, let me say, that the women knows the man is maybe sleeping with other women outside the marriage, and she feels he is going to expose her to infection, but then when she talks about it, he gets rough, he beats her up, maybe he slaps her… Then other times you find… the lady sleeping with the man because she is actually scared of him. Maybe he is her boss at work, maybe the what? Polygamous family, he is one of the big people, maybe an uncle and she is the daughter and she feels like he can easily what? Get physical with her, maybe he can deny her some of the basic requirements.” *Health worker 54-04-2-002, male.*

Health workers reported that specific contextual factors of the refugee settlement such as financial hardship and prevalent substance abuse fuel domestic disputes and aggravate violence.

“They do have the violence at home. Of course, most of the things is associated with money and food… Maybe in refugee side you know, they don’t have any other income generating activity so the only thing they do when they have received maybe this small food… a man goes to sell and… ah the man ends up maybe drinking the money. So, on reaching the woman, find it is now causing violence.” *Health worker 54-07-2-001, male.*

“Alcohol is a problem… If they drink too much, there is violence. No food at home, you cannot pay your child school fees, no clothing, the violence will automatically be there. They say when the poverty enters through the door, then the love jumps through the ventilator… For you when you are drinking, at times you don't [focus on] getting… money for the family.” *Health worker 54-09-2-001, female.*

In these volatile conditions, disputes quickly escalate into violence, especially in those relationships where IPV has occurred in the past. Health workers explained it is important to screen index clients for a prior history of IPV before carrying out APN to gauge the risk of violence occurring following notification.

“Before you consider to do APN, because APN is more of home or community based, there is a need and we assess if there is a possibility of any GBV resulting from the services you [will be] taking to the community.” *Health worker 54-09-2-002, male.*

Health workers suspected that although prior history of IPV is screened for, it is likely underreported by index clients as culture and gender norms make it difficult for clients to disclose such history.

“Usually people find it [a history of violence] very hard to disclose… because they think you want to take any other legal things against them, so they fear disclosing whether they had issues of violence with this partners, or what. Most times they will say no, they don't.” *Health worker 54-05-2-001, male.*

Underreporting of prior IPV history may also be because index clients likely do not recognize forms of violence other than physical violence as IPV.

“Sometimes like where you find like maybe a man has quarrelled at a woman, you find them taking it normal, not knowing that this is one of the violence.” *Health worker 54-07-2-001, male.*

When a history of IPV is present, health workers explained that this issue needs to be addressed before APN can be safely carried out.

“We first deal with the violent part of it, before we bring in testing… so what we do, we invite them here or we do community forum where we visit them at home, and we try to settle the issue. So, once we find that now they are okay, now we intervene in with assisted [partner notification].” *Health worker 54-07-2-001, male.*

#### Interpersonal violence influences notification option choice

For index clients with a history of IPV, health workers reported promoting the choice of a notification option that will minimize risk of additional violence to the index client. A male interview participant (54-02-2-003) stated, “*Because we also want to be sure of the safety of this person… If they say yes [there is a history of IPV], we stop them from self-disclosure. It should now be the provider-initiated.*” Ultimately however, health workers felt that the choice of notification option lies with the index client who knows the partners best.

“Those threats *[of a violent reaction]* have an issue in the type of disclosure they will choose. So most times, when they know, maybe if it is a woman, when she knows that the husband is a hostile person… the fear would be there to disclose. So most times, they would choose the maybe you as health providers you go disclose to what? The person and do your role.” *Health worker 54-05-2-001, male.*

Generally, health workers explained, index clients’ fear of the potential negative consequences associated with sexual partners learning that they may have exposed them to HIV result in a preference for notification options involving the health worker.

#### Interpersonal violence following notification

Interview participants reported that although interpersonal violence following HIV disclosure is feared, it occurs infrequently. The incidents health workers described included physical, sexual, and psychological violence as well as deprivation and neglect. Incidents were of varying severity, with cases of physical violence ranging from quarrelling and fist-fighting to stabbing and in a number of cases even murder.

“It was self-disclosure… the husband [Ugandan national] tested first, then he went home, picked his wife, tested her, she was also positive. Then they went back. The health workers thought it's okay, everything is okay. They went back. At night, he murdered the wife, he murdered the kids… and then he hung himself.” *Health worker 54-04-2-002, male.*

The examples of psychological violence that health workers described included verbal abuse of index clients and forced isolation where index clients were made to eat and sleep in separate rooms away from the rest of the family after their HIV status became known. In some cases, index clients were deprived of basic necessities following notification or were shunned from the community. In the examples health workers described, perpetrators were not limited to sexual partners but frequently included relatives, second spouses or community members. Victims of post-notification violence also did not remain limited to index clients but in some cases extended to dependents as well.

“*[Refugee family]* This woman got married to this man when she is already positive living… *[and]* the child she has *[with her previous husband who died of HIV]* is already positive living. So, that man *[current husband]* came to realize that… the child… is already positive living. So, this man decided… this woman is the one who infected him, actually it is not this woman, it is that husband who has died… who has infected this woman to *[now]* come and… infect [him]. So, because of that *[the current husband]* was hitting that *[deceased husband’s]* child… Stopping this woman to collecting drugs for this child. Not giving that child the drugs. Sometimes even denying this child from right from eating food. So, the child got malnourished… and passed on.” *Health worker 54-07-2-002, male.*

#### Health workers experience violence conducting APN program activities

Several cases were described in which health workers were threatened or attacked while performing APN activities. According to the interview participants these incidents often involved index clients or sexual partners from the South Sudanese refugee community.

“For the refugees it is very difficult. Like my colleague last time tested a refugee positive… She *[the refugee]* doesn't want us to follow her… If you have stayed with the refugees like the Dinka, the Nuer, they are always very aggressive. If somebody says no and you still insist, they can harm you… A colleague it happened one time… They were like chasing her with even a Panga knife… They do not want themselves to be exposed to other people, because we are health workers, people within the communities know that, so, they feel that once we started following her, people are going to get concerned *[will think she has HIV].*” *Health worker 54-05-2-002, male.*

Out of fear of such incidents of interpersonal violence, health workers sometimes decided not to follow up certain clients in the community.

“So, we also take security precautions. Once somebody says no, for them *[the Dinka or Nuer tribe]* we take it as a no *[and no longer try to contact and test them in the community]*.” *Health worker 54-05-2-002, male.*

#### Cultural practices and beliefs contributing to disclosure-related violence

Health workers believed that the cultural practices and beliefs of certain refugee groups from South Sudan play a role in violent reactions to notification of HIV exposure. These South Sudanese tribes lived isolated from other communities in Sudan and health workers believe that as a result, these refugees have had less exposure to HIV and HIV awareness campaigns and interventions. Interview participants explained that this has led to the persistence of misconceptions about HIV and has caused some South Sudanese refugees to believe HIV is strictly a Ugandan disease contributing to hostile reactions when a member of their tribe is notified of exposure to HIV. Among the South Sudanese tribes, health workers identified two tribes – the Dinka and the Nuer tribe – as notably rigid in their beliefs.

“HIV is not, is not a Sudanese disease… They believe they cannot get it. Any person who has been diagnosed with HIV it is even not easy to disclose, to open up… Because if you disclose, definitely you are going to be abandoned… you will not interact with the community… Because from them, they believe you might have messed out *[have had sexual relations with somebody from outside the community, a Ugandan]*.” *Health worker 54-01-2-003, male.*

“South Sudanese, they are known to be a hostile community… like Nuer culture or Dinka culture, if a girl is found positive before marriage, that is a disgrace to the family. So, if you test a Dinka lady positive, she may not allow you to let the family know or any even… Sometimes when they open up, they are disowned by their own communities, so that one brings a challenge, becomes hard.” *Health worker 54-09-2-002, male.*

Interview participants described how the Dinka and Nuer tribes continue to practice traditions such as paying a bride price before marriage which poses a problem when HIV is present. Health workers explained that the sum that families expect to receive for their daughters when they marry causes girls to be regarded as part of a family’s wealth.

“The communities of the Dinka's and Nuer's they are cattle keepers. And when they have a girl child in the home, they look at the girl child as the wealth of the next day or the next tomorrow.” *Health worker 54-09-2-002, male.*

A positive HIV status, interview participants reported, is considered a threat to this wealth as it devalues a girl’s worth. As a result, HIV status disclosure can lead to fighting among families.

“If they identify that this is a young positive lady or a man, and they have the proof, all they know is that this person is HIV positive, and maybe they realize is in a relationship with someone who they know, the community may turn violent against that person because they think he wants to kill the young daughter, or the young girl, and he is being HIV positive… So, they have their local charges, and they will charge you *[request compensation]* because you want to spoil their girl.” *Health worker 54-09-2-002, male.*

#### Blame plays an important role in post-notification violence

Violence, health workers reported, is frequently connected to blame, with the dominant perception in the community being that whoever tests positive first is the one to blame for bringing the infection into the relationship. The reason for this, interview participants explained, is that testing voluntarily is considered to be a sign of a guilty conscience. Despite the availability of ART and improvement in HIV prognosis, some sexual partners continue to regard a positive HIV diagnosis as a death sentence. Exposing someone to HIV is referred to by the community as “killing” someone, and is therefore seen as grounds for violent retribution.

“The lady *[Ugandan national*] went and tested. She tested positive… She said she is going to bring her husband to the facility for testing herself *[assisted disclosure as she would not tell him her status until they both tested together at the facility with a health worker]*… *[so she]* talked to the husband… he refused to come with her to the health facility. But I think deep down, he knew he was messing up somewhere *[knew he had been displaying risk behaviour]*, so he went to a what? To a private clinic and tested alone… They told him he's positive. So, he goes back home, and he asks the partner, ‘When did you test? Have you tested?’ The woman is like, ‘Yes, I have tested’… when he found out that she, she knew her status before and she didn’t tell him and she just asked him to get tested, he took it like she is the one who has actually what? Infected him. Because she was tested positive earlier before him… So, when she admitted, he actually became violent and he got a machete and he chopped her up… He killed her, unfortunately.” *Health worker 54-04-2-002, male.*

Blame related to HIV exposure is complicated in this setting by the practice of polygamy, the cultural custom for men to have multiple wives. Polygamous marriages are common in Uganda [65] and are especially prevalent among the Muslim population in West Nile [66]. In these polygamous relationships, when a woman is notified by her husband of possible exposure, suspicion quickly falls on the co-wife as the source of the infection contributing to disclosure-related violence between co-wives.

“A man has two women… *[One of the women he has]* stayed with seven years… *[The]* first two children *[with this woman]* they were all negative. But now, when the woman got the third pregnant, she came for ANC *[antenatal clinic]* to the outpost there and was tested, now HIV positive… *[When she]* was told that *[she]* is HIV positive, directly went direct and started fighting the co-wife… And said… ‘*[I have]* stayed with my husband for the seven years when you were not yet married… Our two children… were tested negative… you are just being married newly, this is your sixth month in this marriage, *[why is it that now]* I am positive? That means you are the one who has brought the infection.’” *Health worker 54-07-2-002, male.*

To avoid being blamed for infecting their sexual partners, some index clients choose to hide their diagnosis from their partners and decide to take their medication in secret. Health workers reported that when their positive HIV status does come to light, their concealment and the feeling of deception it invokes in the sexual partner can contribute to a violent reaction.

“The man was the index client, and he was on ARV's [*ART]* for some time… But he had never disclosed even to the wife… So we helped… inviting them to the facility, and we assisted him, we assisted him to disclose… Then after one month, so once when the man now felt that maybe everything is now okay, so now the man has again revealed that he had been on ARV's for some time… So, on knowing that, then *[the wife started]* some fighting at home, like how could he hide the information all along? That maybe the man's intention was to infect her. So, it was some fight, serious fighting.” *Health worker 54-07-2-001, male.*

### Convergent mixed-methods findings

Quantitative data from APN registers were compared to and analyzed with findings from qualitative interviews with health workers. The fear that index clients had to list sexual partners as described by health workers was supported by the low average number of sexual partners listed per index client in APN registers. Interview participants attested to routinely screening index clients for a prior history of IPV before performing APN, a statement supported by APN register data that index clients were asked about prior history of IPV for most sexual partners. The recorded prevalence of a positive prior history of IPV in APN registers was thought to be falsely low by interview participants as a result of underreporting given cultural beliefs surrounding acceptable relationship dynamics between index clients and their partners. A disconnect between quantitative and qualitative findings was found regarding screening for post-APN violence. Though health workers endorsed screening index clients for post-APN violence following notification, data on post-APN violence was lacking for most sexual partners in the APN registers suggesting that health workers either did not ask index clients about post-APN violence or did not record their answers. Less than ten incidents of post-APN violence were reflected in the APN registers, but interview participants listed numerous examples of disclosure-related interpersonal violence. Though interview participants specifically named a number of South Sudanese tribes in relation to incidents of violence, APN register data showed similar representation of refugees and Ugandan nationals for the cases of post-APN violence.

## DISCUSSION

APN services at health centers in or near refugee settlements serving refugee and national populations in West Nile Uganda have contributed to notifying sexual partners of index clients of possible HIV exposure. Health workers involved in HIV testing and counseling services list multiple examples of physical, sexual, and psychological violence as well as deprivation and neglect following HIV disclosure and qualitative interviews suggest that post-APN violence strongly influences index clients’ participation in APN. However, data on post-APN violence are lacking in APN and HIV registers suggesting that index clients are rarely screened for post-notification violence or that outcomes are rarely reported. For the minority of cases in which screening was recorded (20%) only 8 incidents of post-APN were reported since initiation of APN services.

Qualitative interviews highlight the ubiquitous presence of interpersonal violence in West Nile refugee settlements. Health workers explain that challenging economic circumstances in the refugee settlements, and prevalent substance use [67,68], and cultural norms regarding male-female relationship dynamics contribute to a volatile atmosphere in which violence easily erupts. Although strides have been made in HIV education and awareness in Uganda [69-71], health workers interviewed in this study report that when a person exposes someone to HIV in this setting, this is still referred to as “killing” someone by the West Nile community. This continued perception of HIV as a death sentence makes disclosure of possible HIV exposure a dangerous affair.

We aimed to elucidate the role of interpersonal violence in APN, looking specifically at how interpersonal violence influences APN utilization and the occurrence of post-APN interpersonal violence. Health worker interviews indicate that violence and fear of violence strongly influence APN utilization, contribute to underreporting of sexual partners, affect notification option choice, and alter program execution by health workers. These findings are in accordance with previous studies looking at partner notification services that identify fear of the repercussions of notification in general and fear of violence in particular, as major barriers to APN participation for index clients [5,22,41]. Interviews with health workers highlight that fear of violence is experienced differently by men and women, a discrepancy stemming from gender roles and inequalities in relationships that have previously been shown to impact decisions surrounding HIV testing and disclosure [5,72-77].

For index clients in West Nile, as was observed previously by Monroe et al. in Kenya, fear of violence is not directly linked to participation in partner notification services but rather to the broader fear of others becoming aware of their HIV status [41]. This fear persists despite availability of anonymous notification options suggesting that index clients lack trust in program confidentiality, a challenge that has been identified in other settings where partner notification services have been implemented [41,78]. Guaranteeing confidentiality of APN is complicated however, by standalone HIV clinics at the health centers at which attendance is very visible and by the necessity of involving third parties such as translators in APN. Even when confidentiality is strictly adhered to by all parties involved, anonymity of the index client cannot be guaranteed in monogamous relationships where HIV exposure can only be traced back to one person putting these index clients at risk of disclosure-related violence.

Consistent with prior evidence [31,79], qualitative interviews reflect that health worker-mediated forms of disclosure such as assisted and provider notification are preferred by and encouraged for index clients who fear a violent response from sexual partners as these forms are thought to protect against post-APN violence. Past research has shown that index clients with prior exposure to violence are at increased risk for post-notification violence [11,31,32]. The current study shows that these index clients with prior history of violence make significantly different notification option choices than index clients without prior exposure to violence. The extent to which this divergence results from counseling towards certain notification options by health workers or stems from a personal preference is unknown. Surprisingly, in the current study, a prior IPV history was found to be associated with an increase in the percentage of index clients opting for assisted notification – a notification option in which index clients maintain the opportunity to first attempt to notify partners themselves, as opposed to provider notification in which notification is performed directly and anonymously by the health worker, an aspect that might be expected to be beneficial. This may be because participants with prior IPV exposure wish to retain control over disclosure but at the same time recognize the safety benefit of having a health worker present to help prevent a response of sudden violence.

When asked about interpersonal violence following notification through APN services, health workers give detailed accounts of cases of physical, sexual, and psychological violence as well as deprivation and neglect. The lack of data on post-APN IPV recorded in APN registers however suggests index clients are either rarely formally screened for post-APN violence or that health workers are not compelled to record the result. With these missing data, the safety of APN participation in refugee settlements in West Nile remains insufficiently evaluated. Limited quantitative data on post-APN violence hamper the identification of index client and sexual partner characteristics that significantly predispose to post-APN violence. The presence of a history of IPV in all but one of the 8 post-APN IPV cases supports the notion that index clients with a history of IPV are at a higher risk for disclosure-related violence. The ability of interview participants to list multiple cases of post-notification interpersonal violence appears at odds with previous APN studies that describe social harm as a rare result of partner notification services [2,4,6-9,20-25,30]. This discrepancy warrants further study but may be partly attributable to context-specific factors including the high prevalence of prior exposure to violence seen in refugee settlements, cultural practices and acceptance of GBV observed in the West Nile region, as well as the procedures for screening and registration of violence in the current study and previous partner notification studies. The cases of violence against health workers as they carry out APN activities that came to light in this study have not previously been described and also warrant further examination.

In qualitative interviews, health workers identified specific South Sudanese tribes as frequent instigators of disclosure-related violence in the refugee settlements. Interview participants believed these hostile reactions to be the result of a lack of exposure to HIV awareness activities and differences in cultural norms and customs. No statistically significant difference however, was found in representation of Ugandan nationals and refugees for the cases of post-APN IPV recorded in APN registers suggesting that health workers, who generally are Ugandan nationals, may possess a certain degree of bias towards these foreign tribes. This is in line with previous reports from refugee settlements that describe stereotyping by Ugandans of particular refugee groups who live further away from the Ugandan border and display less cultural similarities to Ugandans [80]. Among these refugee groups are the Dinka and Nuer tribes that are mentioned in the qualitative interviews. Ugandan host communities have been reported to accuse these groups of arrogance and violence and to describe these tribes as generally having “a difficult temper” [80].

The consistent success of APN services across SSA in identifying and diagnosing individuals exposed to HIV emphasizes the program’s potential and supports the recommendation of the WHO to offer these services as part of a comprehensive package of testing and care [81]. If partner notification services are to be widely implemented however, the safety of these services needs to be further investigated. Previous studies examining social harm following APN have not monitored for violence perpetrated by family and community members [2,4,6-9,33] – social groups demonstrated in this study to be highly involved in cases of interpersonal violence following APN. Prior studies have also excluded certain groups such as index clients with a (recent) history of IPV who are at a high risk of violence following HIV disclosure from study participation [4,22]. Exclusion of certain groups from participation in partner notification services however, is not stipulated in the WHO guidelines on HIV self-testing and partner notification [1]. Rather, WHO guidelines recommend assessing risk of harm on a case-by-case basis in consultation with the index client. Safety of the program is promoted by ensuring it is voluntary and confidential and by recommending screening for IPV, counseling on different disclosure options and possible outcomes, and by making sure relevant referral resources are in place [1]. Similarly, in Uganda, the Ministry of Health guidelines on APN do not exclude those with a history of violence from APN participation but do suggest that if index clients disclose prior IPV with a sexual partner, notifying this partner may not be appropriate unless the client’s safety can be assured [54]. Interviews with health workers in this study reveal that in practice, in Uganda, participation in APN and notification option choice are left up to the index client’s discretion. While health workers describe advising index clients with prior history of IPV to opt for assisted or provider notification, this is by no means a guarantee of safety as is illustrated by the fact that in this study all incidents of post-APN violence reported in APN registers occurred following assisted and provider notification. Targeted APN adaptation for high-risk index clients is complicated by the suspicion that a prior history of IPV is underreported by index clients. Although HIV disclosure-related violence is not considered a regular occurrence following APN, there are sufficient examples to warrant more rigorous monitoring of violence and further research into the safety of APN.

The findings of this study should be considered in the context of the study’s limitations. Missing data in APN registers limit the validity of the quantitative data and make it difficult to draw conclusions about the scope of interpersonal violence following APN participation. Quantitative and qualitative data were analyzed concurrently, preventing interviews from being adapted throughout the study to explore any disconnect between the two sets of findings. The limited data on post-APN violence recorded in APN registers while interview participants report routinely screening index clients for IPV following notification is an example that warrants further investigation. Interview participants’ inability to accurately recall the timing details of index clients’ interpersonal violence experiences (which occurred with variable timing in relation to the health care encounters) limits the extent to which causal conclusions can be drawn about these incidents in relation to the APN program. For example, some experiences of violence may have been related to HIV-disclosure prior to APN initiation. While health workers play an important role in APN and their views and experiences offer valuable insight into the APN process, the perspective of index clients and their sexual partners – the people who experience interpersonal violence and participate in APN – is vital to understanding how interpersonal violence affects APN utilization. Due to resource and time constraints, assessing this population fell outside the scope of this study.

## CONCLUSION

APN register data and accounts of health workers providing HIV care and APN services in or near refugee settlements serving refugee and national populations in West Nile Uganda reveal that incidents of interpersonal violence occur following APN participation and suggest that interpersonal violence and fear of interpersonal violence influence APN processes. Fear and occurrence of disclosure-related violence are intertwined with cultural perceptions and associations regarding HIV. Future research is needed to prospectively evaluate how prior experiences of violence affect APN participation by index clients and sexual partners and to investigate whether APN is associated with subsequent violence. A better understanding of the role of interpersonal violence in APN is vital to safely engage vulnerable refugee populations in HIV care.
